# Tentaculate Fossils from the Cambrian of Canada (British Columbia) and China (Yunnan) Interpreted as Primitive Deuterostomes

**DOI:** 10.1371/journal.pone.0009586

**Published:** 2010-03-08

**Authors:** Jean-Bernard Caron, Simon Conway Morris, Degan Shu

**Affiliations:** 1 Department of Natural History-Palaeobiology, Royal Ontario Museum, Toronto, Ontario, Canada; 2 Department of Earth Sciences, University of Cambridge, Cambridge, United Kingdom; 3 Early Life Institute and Key Laboratory of Continental Dynamics, Northwest University, Xi'an, People's Republic of China; University of Maryland, United States of America

## Abstract

Molecular and morphological evidence unite the hemichordates and echinoderms as the Ambulacraria, but their earliest history remains almost entirely conjectural. This is on account of the morphological disparity of the ambulacrarians and a paucity of obvious stem-groups. We describe here a new taxon *Herpetogaster collinsi* gen. et sp. nov. from the Burgess Shale (Middle Cambrian) Lagerstätte. This soft-bodied vermiform animal has a pair of elongate dendritic oral tentacles, a flexible stolon with an attachment disc, and a re-curved trunk with at least 13 segments that is directed dextrally. A differentiated but un-looped gut is enclosed in a sac suspended by mesenteries. It consists of a short pharynx, a conspicuous lenticular stomach, followed by a narrow intestine sub-equal in length. This new taxon, together with the Lower Cambrian *Phlogites* and more intriguingly the hitherto enigmatic discoidal eldoniids (Cambrian-Devonian), form a distinctive clade (herein the cambroernids). Although one hypothesis of their relationships would look to the lophotrochozoans (specifically the entoprocts), we suggest that the evidence is more consistent with their being primitive deuterostomes, with specific comparisons being made to the pterobranch hemichordates and pre-radial echinoderms. On this basis some of the earliest ambulacrarians are interpreted as soft-bodied animals with a muscular stalk, and possessing prominent tentacles.

## Introduction

An understanding of the Cambrian “explosion” arguably provides one of the best avenues to tackling two major questions in evolutionary biology: How are metazoan bodyplans assembled and what, if any, role do macroevolutionary processes play? In addition, this event provides a crucial test as to the relevance of the fossil record either to decide between conflicting phylogenetic hypotheses or to determine the actual course of events. Given the crucial contributions of the fossil record to understanding such key episodes as the terrestrialization of the sarcopterygian fish or the capacity of theropod dinosaurs to engage in flight, then it might seem paradoxical that equivalent material is of ambiguous status when it comes to the interpretation of the Cambrian “explosion”. The reason largely revolves around the fact that a significant number of these early animal fossils have very unfamiliar, if not bizarre, anatomies, exhibiting a combination of characters not encountered in any extant phyla [1], [2]. Traditionally regarded as Problematica (i.e., of unknown affinity), these bizarre taxa present a significant challenge in terms of often complex preservation, not to mention the difficulties in reconstructing a plausible functional morphology.

What then is the best line of approach when it comes to establishing their evolutionary position? One procedure is to treat these ostensibly strange fossils as extinct bodyplans. In the literature these are often equated with the level of phylum, and the idea of a plethora of extinct Cambrian phyla has played a significant role in evolutionary interpretations of the Cambrian “explosion”[3]. The clade we identify here has considerable disparity, ranging from pedunculate to discoidal animals, but we argue still represents a distinctive and identifiable bodyplan whose wider relationships are less easy to establish. Should we designate a new phylum? In principle the taxon Dendrobrachia [4] is available, but the diagnosis by X-G. Hou *et al.* only encompasses one of the taxa (*Phlogites*) that we consider here. Another important consideration is that the cladistic methodology attempts to identify stem-groups, and this has important strengths in understanding how bodyplans might be assembled as against the more essentialist stance of phyla. Accordingly, rather than expanding the concept of Phylum Dendrobrachia (and recalling also that the rules of priority laid down by the ICZN do not extend to the taxonomic level of phylum), here we employ the informal stem-group category of cambroernids whose currently recognized taxa include *Phlogites, Herpetogaster* and all members of the eldoniids (including *Eldonia*, *Paropsonema*, *Rotadiscus*, and *Stellostomites*).

It is important to recall that not only is the concept of phyla essentialist but it serves to place problematic taxa in a phylogenetic limbo, rendering them effectively immune to further evolutionary analysis. An alternative strategy is to assign a supposedly enigmatic taxa to one or other extant group, that is somewhere within a given crown-group. This, however, is often a procrustean process. This is because it generally relies on comparisons between various structures in the fossil material (the preservation of which may be less than perfect) and extant material, a procedure that may be based on pre-suppositions that are not necessarily spelt out unequivocally. So too by placing these enigmatic Cambrian taxa in crown-groups, it can presuppose a deeper origin of phyla than appears to be consistent with the fossil record. A third way is to assign these fossils to various stem-groups of either a major phylum (e.g., an echinoderm) or some more embracive super-clade (e.g., the ambulacrarians). This procedure has, however, its own difficulties. This is because of the need to homologize morphologically disparate structures, a procedure which is effectively the converse of attempting to accommodate these taxa in crown-groups. The advantage of this approach is that it has the potential to explain how key characters evolved in the emerging bodyplans within a functional and ecological context. These characters can then be placed in a series of paraphyletic stem-groups [1].

In principle, by testing various evolutionary scenarios a cladistic methodology will be able to impart a rigorous approach when dealing with the evolution of bodyplans and the interpretation of Problematica. In practice, however, this ideal remains somewhat elusive. The fact remains that the available fossil record is very patchy and as often as not, there is a dearth of very well-preserved material and characters available.

**Figure 1:**
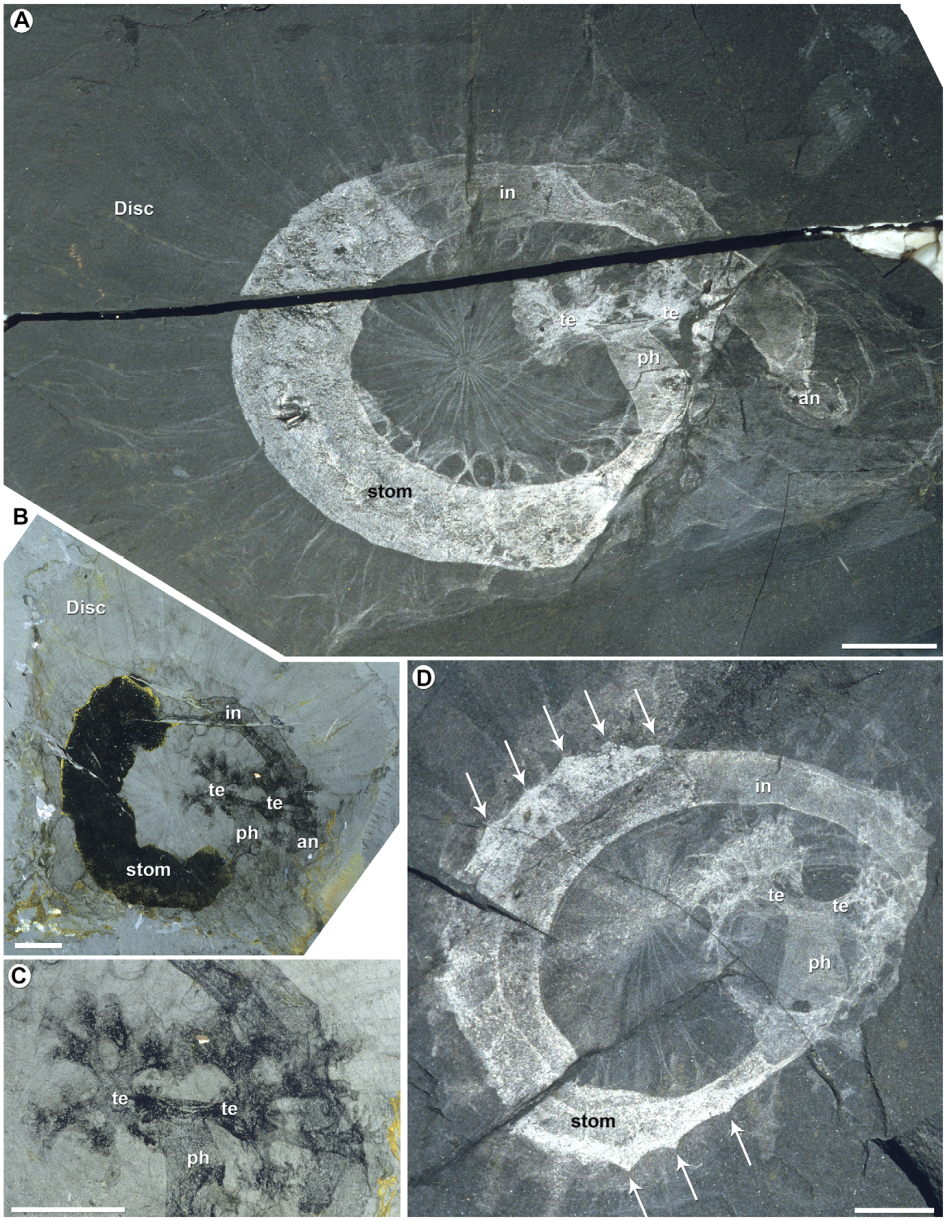
*Eldonia ludwigi* from the Middle Cambrian Burgess Shale. A, lectotype, National Museum of Natural History (USNM) 57540 Part, overall view. B–C, USNM 188552; B, Part, overall view; C, detail of the tentacles. D, USNM 201692 Part, overall view, arrows pointing to triangular projections representing evidence of possible segmental mesenteries. Scale bars: 10 mm. an, anus; in, intestine; ph, pharynx; stom, stomach; te, tentacle. (Photos D. Collins).

Despite these difficulties there does appear to be some progress in the identification of metazoan groups amongst various Cambrian taxa. Here we aim both to extend this process, but also provide a model example of the difficulties that emerge as the comparisons are extended to higher taxonomic levels. In brief, on the basis of conspicuous morphological similarities we demonstrate that a new soft-bodied fossil from the Middle Cambrian Burgess Shale (*Herpetogaster collinsi*) is related to several well-known fossil Problematica, specifically a group known as the eldoniids [5]–[10] as well as the taxon *Phlogites longus* [4], [11] from the Chengjiang Lagerstätte of Yunnan, China (the poorly known and coeval *Conicula* [11] also appears to be a phlogitid). In terms of affinities *Phlogites* has been compared to the gnathiferans [4] (albeit including various taxa that are not currently members of this group [12]), placed within its own phylum Dendrobrachia [4], or alternatively interpreted as a lophophorate [11], [13], or a stem-group lophophorate [14]. The new fossil material presented in this study lends no support to any of these proposals. Whilst a connection between this taxon and *Herpetogaster* seems unproblematic, the inclusion of the eldoniids is more significant. This is because although this group has a broad geographic and stratigraphic distribution (which extends significantly beyond the Cambrian, although the many exceptionally preserved specimens are largely known from various Lower and Middle Cambrian fossil Lagerstätten [5], [10], [15]), the wider affinities of the eldoniids have remained highly controversial. Thus, suggestions have covered the spectrum of metazoan affinities, with the eldoniids being placed within the cnidarians, as well as the deuterostomes (as holothurians) and protostomes (as lophophorates). With a seemingly highly distinctive bodyplan characterized by a conspicuous disc with a variable number of radiating lobes and a pair of oral dendritic tentacles (Figure 1) the eldoniids exemplify the central problem of interpreting early metazoan evolution in the context of a plethora of bizarre fossil taxa.

*Herpetogaster* helps, therefore, to bridge the gap in morphology between a number of seemingly problematic forms. In recognition of this we thereby identify this taxon, *Phlogites* and the eldoniids as belonging to a clade informally identified as the cambroernids. The available characters suggest that taken together a position of this clade within the deuterostomes, and specifically the ambulacrarians, is the best current phylogenetic solution. Nevertheless the paucity of useful characters and the possibilities of convergence make this assignment tentative and further progress will depend on the discovery of new fossil taxa.

## Results

### (a) Systematic Palaeontology

?Superphylum Ambulacraria

#### Unranked stem-group

Cambroernids, in reference to the stratigraphic age and prominent branched tentacles (Greek, *ernos*), see above for discussion of Phylum Dendrobrachia [4], which was erected for a single species (*Phlogites longus*).

#### Diagnosis

Metazoans with prominent feeding tentacles and conspicuous gut housed in a coiled coelomic sac suspended by mesenterial elements, body form ranging from pedunculate to discoidal.

*Herpetogaster* gen. nov.

urn:lsid:zoobank.org:act:F9124AEB-1937-4570-881D-6068AE78A62E

#### Type species

*Herpetogaster collinsi*.

#### Other taxa

*Phlogites longus* Luo & Hu 1999 [11]; for synonymy list see Hou *et al*. [4], *Conicula striata* Luo & Hu 1999 [11]. Note some authors [e.g.], [4,13] have also included *Cheungkongella* as a junior synonym of *Phlogites* rather than a distinct taxon related to the tunicates [16]. No part of this paper depends on the correctness of either interpretation, and it seems more prudent to leave both taxa separate at this time. The only known specimen of *Cheungkongella* is similar to *Phlogites* only in its lower part. The upper part shows a single distinct oral siphon with short simple tentacles which do not compare well with the five large branching tentacles present in *Phlogites*.

#### Etymology

The genetic name refers to the collectors' nick-name “creeposaurus” [creeping aspect of the animal] (Greek, *herpeto*) and the prominent stomach (Greek, *gaster*). The species name honours Desmond Collins, leader of the Burgess Shale Royal Ontario Museum (ROM) expeditions (1975 to 2000), when the first specimens were collected.

#### Holotype

ROM 58051. 100 additional specimens: ROM 57164, 57167, 58022-58090, 58158-58160, 58928-58930, 59850, 59852-59854.

#### Stratigraphy, referred material and locality

Burgess Shale and Stephen Shale Formations; Middle Cambrian, Yoho and Kootenay National Parks, British Columbia, Canada.

#### Diagnosis

Segmented body, coiled dextrally. Short head bearing prominent anterior dendritic tentacles and pharyngeal structures, possibly lateral pores. Trunk, sub-cylindrical, divided into two sub-sections, narrowing posteriorly. Ventral and contractile adhesive stolon, sometimes with terminal disc. Digestive tract with anterior mouth, pharynx, voluminous stomach, and narrow intestine with terminal anus. Stomach and intestine of sub-equal lengths, un-looped, with triangular mesenterial insertions.

*Herpetogaster collinsi* gen. et sp. nov.

urn:lsid:zoobank.org:act:2759BD4E-6ACA-4C5D-92A2-29DF511E3071

#### Diagnosis

Head with bilateral tentacles in basal two plus two arrangement, sub-equal length, monopodial, fractal-like branching. At least 13 trunk segments, well developed on dorsal anterior.

#### Remarks on preservation

**Figure 2:**
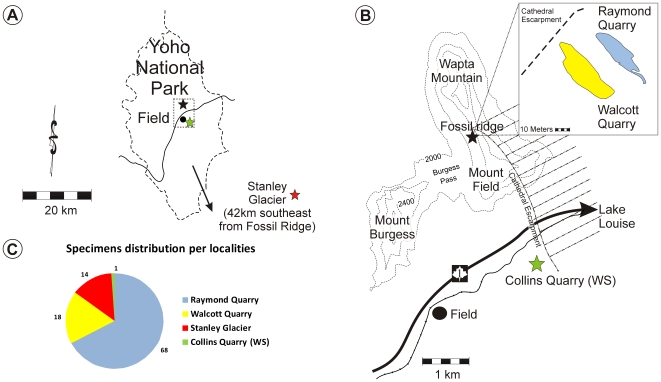
*Herpetogaster collinsi* from the Middle Cambrian Burgess Shale. A–B, Locality maps. C, specimen occurrences.

Whilst we present evidence for a relationship between *Herpetogaster* and the eldoniids, the simpler hypothesis that the former taxon could represent a preservational variant of *Eldonia* can be firmly rejected. With two exceptions (dissociated tentacles), all specimens seem to have been complete at the time of burial. Overall, a range of minute preservational differences can be recognized from perfectly distinct body outlines with well preserved tentacles and clear segmental boundaries to poorly preserved specimens showing evidence of decay of the tentacles still being in contact with the trunk. There is no evidence of tearing of tissues which could indicate decay of a dorsal disc, this being one of the most conspicuous body elements in *Eldonia* and other Cambrian discoidal forms (Figure 1). Discoidal animals related to *Eldonia* from the Chengjiang and Kaili biota [5] show clear evidence of decay of the tentacles as in *Herpetogaster* but retain clearly the dorsal disc, presumably because this disc is more resilient to decay than the tentacles. *Herpetogaster* occurs in 11 bed assemblages from the Walcott Quarry [17] and 20 bed assemblages from the Raymond Quarry [18] in addition to two other localities (Figure 2). Patterns of co-occurrence of the two species provide additional support for separating both taxa; both species only co-occur in the Walcott Quarry in three bed assemblages.

#### Description

**Figure 3:**
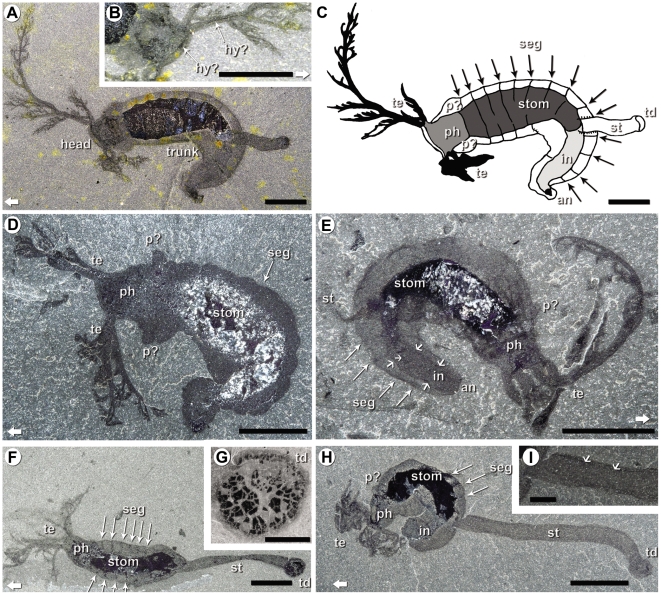
*Herpetogaster collinsi* from the Middle Cambrian Burgess Shale. All specimens are preserved dorso-ventrally, except F. In each photographic figure anterior is indicated by a wide white arrow. A–C, Royal Ontario Museum (ROM) 58051 Holotype. A, Part, overall view; B, Counterpart, detail of tentacles with hydrostatic canal and/or vascular system emphasized by white arrows; C, Camera-lucida drawing of part and counterpart emphasizing the presence of putative segment boundaries and triangular projections along the stomach. D, ROM 58046 Part, with symmetrical tentacles and pharyngeal pores. E, ROM 58039 Counterpart, intestine with putative enclosing tube emphasized by small arrows. F, G, ROM 58037 Part. F, Part, lateral view; G, Detail of terminal disc. H, I, ROM 58047 Part. H, extended stolon and terminal disc; I, Detail of the stolon, small arrows point to a darker central area representing a possible coelomic cavity. Scale bars: A–F, H, 5 mm; G,I, 1 mm. an, anus; hy?, putative hydrostatic canal and/or vascular system; in, intestine; p?, putative pharyngeal pores; ph, pharynx; seg, segment boundary?; st, stolon; stom, stomach; td, terminal disc; te, tentacle.

**Figure 4:**
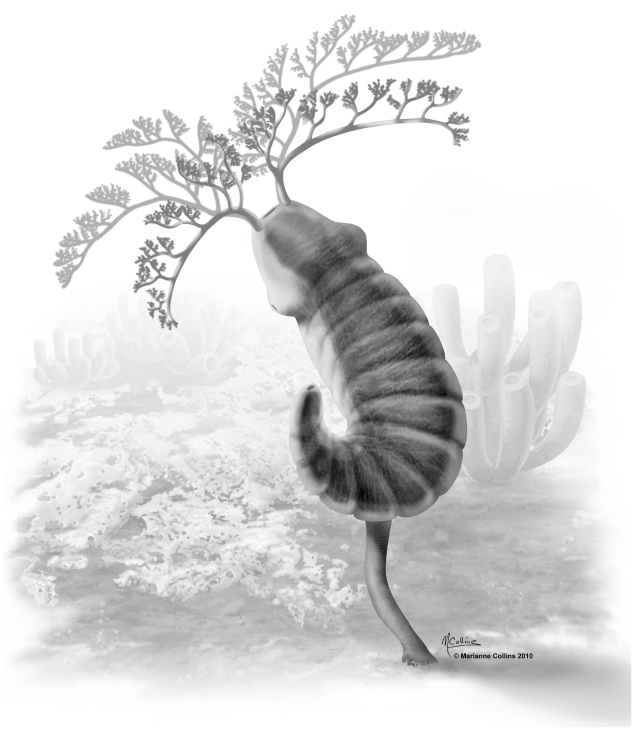
Reconstruction of *Herpetogaster collinsi* from the Middle Cambrian Burgess Shale. © 2010 - Marianne Collins.

The body of *Herpetogaster* consists of a head with tentacles, trunk, and stolon (Figures 3, 4). Its bilateral symmetry is most clearly defined by the paired tentacles (Figure 3D) and lateral lobes along the head (Figure 3A, C, D), whilst the more developed banding, on one side of the trunk is tentatively regarded as dorsal. The re-curved trunk has a terminal anus (Figure 3E), and a stolon arises from the ventral mid-trunk, sometimes terminating in an attachment disc (Figure 3F–H). Viewed dorsally the trunk invariably inclines clock-wise combined with a modest helical translation (Figure 3A, C, D, H). The body is not mineralized and was evidently encased in a fairly tough but flexible integument. Including tentacles, specimens are typically 3–4 cm in length (allowing for curvature, see Figure S1).

The tentacles (Figure 3A, E) emerge from the corners of the head (Figure 3A–F, H), and were evidently softer than the rest of the body. On either side they appear to arise as a pair, although it is possible that each inserts via a single stalk which then immediately bifurcates (Figure 3B, C). The tentacles tend to vary in length within and between pairs (Figure 3A). In some cases they are clearly asymmetrical, but other specimens have tentacles of roughly similar sizes (Figure 3D, H). Length variations are probably controlled in part by both angle of burial and decay (Figure 3A), but like the stolon, the tentacles were probably extensible. Branches usually face inwards, and their overall arrangement is indicative of a monopodial growth (Figure 3A–D). At least five orders of branching are recognized, with branch diameter decreasing distally (Figure 3A, C). The medial zone typically houses a narrow dark strand (Figure 3B), connecting to a darker zone inside the head (Figure 3B). The style of preservation (darker and more reflective like the gut) and topology suggest this structure is probably internal and might represent either a hydrostatic canal and/or vascular system; it is less likely to represent an external feeding groove.

In dorso-ventral specimens the frontal margin of the head is straight and broad (Figure 3A–D), but in lateral view (Figure 3F) it appears narrower suggesting the head had an elliptical cross section. In some dorso-ventral specimens conspicuous hemispherical to semi-triangular structures (occasionally duplicated on at least one side) extend from either side of the posterior head (Figure 3A–E, H). Their function is not known, but their prominence and bilateral arrangement suggest they could have an association with the pharynx representing potential pharyngeal pores. The trunk shows transverse bandings interpreted as segments and numbering up to 13. They extend from the level of the anterior margin of the stomach to the posterior tip, and impart a distinct scalloped appearance to the body margins (Figure 3A, C, D, F, H). The extension of the segments across the gut (Figure 3A, C) and the presence of darker areas delimiting each segment indicate that these latter structures are unlikely to be internal mesenteries but are integral to the segments. The presence of triangular projections along both sides of the digestive tract, however, is interpreted as possible insertion points for mesenteries that in both directions would have suspended the digestive tract from the body wall (Figure 3A, C, E).

The mouth is assumed to have been located on the anterior margin and between the tentacles. A central darker area, which in dorso-ventral view occupies much of the head, is interpreted as a pharynx (Figure 3A–F, H), flanked anteriorly by the probable canals/blood vessels. Posterior to the pharynx, the alimentary canal is composed of two distinct parts of sub-equal lengths; a conspicuous lenticular stomach and a straight, un-looped intestine. In dorso-ventral view the stomach occupies much of the trunk (Figure 3A, C–E, H), whereas viewed laterally it is narrower (Figure 3F), suggesting it was compressed in cross-section. The intestine is usually a faintly preserved canal which starts with a slight constriction near the stomach (Figure 3A, C, E). A darker zone on either side of the intestine may represent an enclosing membrane, which also is seen to enclose the stomach (Figure 3E). Darker material at the rear end of the trunk (Figure 3A, C), and sometimes extending to the adjacent sediment, possibly represents gut contents seeping from the anus.

An elongate structure, termed here the stolon, extends from approximately the ninth trunk segment. It evidently arises from the ventral mid-line (Figure 3A, C, E, F, H), and is highly variable in length. Within its length a central dark zone may represent a coelomic cavity (Figure 3I). The termination of the stolon is generally simple, but occasionally bears a dark reflective film with some relief (Figure 3F, G). A mode of life as an epibenthic suspension feeder, occupying the intermediate feeding tier (Figure 4), is indicated by the elongate and dendritic tentacles and the contractile and flexible stolon, and in some cases a defined holdfast. There is no evidence of mouth parts and gut content that would indicate predation on macroscopic animals. Evidence for a hydrostatic skeleton, presumably coelomic, is inferred from structures in the tentacles and stolon, and most likely a similar cavity extended along the trunk. On occasion the animal may have been attached directly to the epibenthic sponge *Vauxia*, with which it is commonly associated. A gregarious lifestyle is indicated by clusters of specimens (up to 8 on the same slab, see Figure S2).

### (b) *Phlogites*

**Figure 5:**
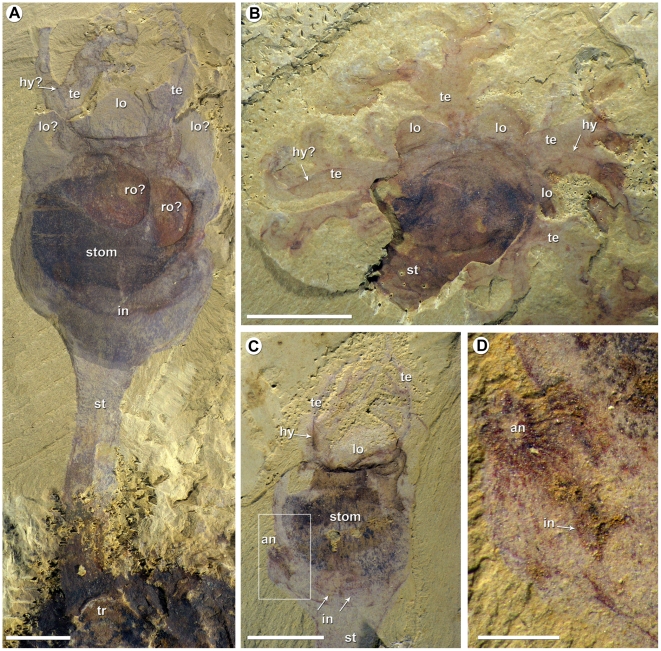
*Phlogites longus* from the Lower Cambrian Chengjiang biota. A, ELI-Phl-07-001 lateral view, complete specimen with the stolon attached to a fragment of trilobite exoskeleton. B, ELI-Phl-07-002 oral view, specimen showing four tentacles (the broken area suggests the possibility of a fifth set) and three preserved lobes. C–D, ELI-Phl-07-003 lateral view; C, overall view of the gut; D, detail of the anus. Scale bars: A–C, 5 mm; D, 1 mm. Legend, see figure 1; lo?, lobes; ro?, reproductive organs?, tr, trilobite fragment.

The somewhat older *Phlogites* [4], [11], [13], [14], [19] is similar to *Herpetogaster*, but shows a number of differences that may reflect an adaptation to a completely sessile mode of life (Figure 5, see camera-lucida drawings, Figure S3). Thus, the stolon is more massive, effectively continuous with the body, and shows an ornamentation of longitudinal strands and fine transverse folds [4]. It appears to be invariably attached to a hard substrate, such as trilobite debris [4] (Figure 5A) or vacated tubes [19]. The body is more calyx-like, and the posterior region is reduced to a small lobate extension with the anus opening laterally [4], [14] (Figure 5A, C). The arrangement of the gut reflects the configuration of the body, with the oesophagus joining a large quadrate stomach and thence in a clockwise direction leads to a recurved, narrow intestine, sub-equal in length [4] (Figure 5A). The anterior of the body bears prominent lobes [4], each semi-circular in shape with a smooth margin, which are located between the tentacle bases (Figure 5B). The tentacles appear to have had at least four (more likely five) separate insertions on to the rim of the calyx. The tentacles themselves are massive, and show an apparently dichotomous branching with simpler terminations (Figure 5B). Similar to *Herpetogaster*, internal strands, possibly blood vessels and/or coelomic cavities, extend from the tentacles into the anterior body. A characteristic feature of *Phlogites* is two to three ovate structures with some relief within the anterior part of the calyx. They are evidently an integral part of the body, and their original function is speculative; possibly they were reproductive tissue (Figure 5A).

### (c) Eldoniids

The best known eldoniids come from various Cambrian fossil Lagerstätten: *Eldonia ludwigi* from the Burgess Shale (Figure 1) and *Stellostomites eumorphus*, *Rotadiscus grandis*, and *Pararotadiscus guizhouensis* from southwest China. These various taxa are distinguished on the basis of relatively minor morphological differences of the disc, tentacles and internal radiating lobe structures [5]. In all forms, the mouth and anus open ventrally and off-centre. There is a prominent pair of tentacles, while internally the gut consists of a pharynx, voluminous stomach and narrower intestine, all located in a distinct coiled sac. As with *Herpetogaster* and *Phlogites* [13] this has a clockwise orientation. Whilst often interpreted as pelagic filter feeders [10], the eldoniids have been reinterpreted [15] as benthic deposit feeders, with the tentacles raised above the sea-floor. An alternative view with the tentacles sweeping the sea-floor is corroborated by the types of depositional environment, patterns of preservation (when recorded in the field most eldoniids are recovered disc-up [5], [6], fossil associations and functional morphology (stiffness of the disc, and evident lack of gas-filled chambers to confer buoyancy). It is less certain, however, whether eldoniids were completely sedentary [15] or had at least a semi-vagrant lifestyle.

## Discussion

### (a) Comparisons between *Herpetogaster*, *Phlogites* and the Eldoniids

The overall similarities between *Herpetogaster* and *Phlogites* strongly suggest a close relationship. Moreover, despite a seemingly disparate anatomy the eldoniids also share important features with these two taxa (see also [4] for a comparison between eldoniids and *Phlogites*). Amongst the similarities considered to be homologous are: a coiled sac that houses a voluminous stomach and narrow intestine, at least one pair of oral tentacles that bifurcate several times along their lengths (eldoniid tentacles are shorter and more robust; tentacles in *Rotadiscus* only bifurcate once at their base [5]). Assuming the arrangement seen in *Herpetogaster* is the more primitive, a transformation to the medusoid-like body of the eldoniids would have been achieved by a lateral expansion of the dorsal and ventral body walls to form upper and lower surfaces of the disc, combined with further clockwise rotation about the helical axis so the posterior intestine came to underlie the oral region. These re-alignments led in turn to a shortening of the tentacles which necessarily needed to remain external, loss of the stolon and development of a complex radial system of mesenteries and canals. The transformation from a worm-like to medusiform organism also explains the radial arrangement of the mesenteries and canals, and for at least the former it seems possible that they originated from the segments in *Herpetogaster* (note these are not evident in *Phlogites*, and presumably were lost as a result of the transformation to a calyx-like arrangement of the body). In any event, these realignments would have necessitated employment of a mesenterial system to anchor the alimentary canal in eldoniids. In this scenario, *Herpetogaster* with its single pair of tentacles might represent a primitive stage leading to forms with a more cup-shaped (phlogitid) and ultimately a disc-shaped body (eldoniid).

Accepting our arguments for significant homologies that unite *Herpetogaster + Phlogites* and the eldoniids (as noted above collectively identified as the cambroernids). Arguably, of course, tentacle morphology and gut anatomy might have more to do with a particular feeding strategy than shared ancestry and could then be interpreted to be convergent. After all, the “Lophophorata” have been united by morphological characters such as possession of tentacles but as a result of recent molecular phylogenies are now thought to represent a polyphyletic group (see discussion below). The broadly similar age of *Herpetogaster + Phlogites* and the eldoniids does, however, go some way to support our argument for this being a monophyletic association. In addition, it is worth emphasizing that no other Cambrian or post-Cambrian fossils display a similar association of characters. Nevertheless in the absence of additional independent data to test our hypothesis, we consider the monophyly of the cambroernids to be a working hypothesis.

### (b) Difficulties in Phylogenetic Interpretation

As noted above in the introduction, the problems of assigning apparently problematic taxa (which are by no means confined to the Cambrian [20]) to known groups remain significant and they apply with particular force to determining the wider affinities of the cambroernids. As already noted the usual route of phylogenetic analysis employing cladistic analysis unfortunately remains problematic. This is on account of the relative paucity of characters, the assignment of morphological features that defy unambiguous interpretation, and crucial decisions concerning characters that might be shared between the cambroernids and a number of quite different major animal groups that remain extant. Comparisons between these fossils and various living forms have the potential advantage of increasing both the number of taxa and characters available for study. As noted, however, the character complex that defines the cambroernids most likely went extinct in the mid-Palaeozoic, so that with approximately 400 Ma separating this clade from the extant faunas the risk of potentially informative characters actually being homoplasious needs to be considered. In addition, classical developmental and morphological characters in extant taxa (such as the fate of the blastopore, cleavage patterns, mesoderm and coelomic cavities) are by no means easy to recognize (or simply unavailable) in many fossils, including the material under consideration here. As importantly many such characters, long thought to be phylogenetic hall-marks for the interpretation of metazoan evolution, are increasingly interpreted as being highly labile and point to the likelihood of extensive homoplasies. Thus, it is widely accepted that the lophophores of some lophotrochozoans are quite independent of the comparable structures in the pterobranchs, even though at a microscopic level they are very similar [21]. Moreover, because of the lack of detailed morphological studies in many groups, especially at the microscopic level, even in extant forms it may be difficult to determine whether or not some characters have evolved relatively independently (e.g., U-shaped gut or tentacles). Owing to these difficulties, we believe that the best way to tackle the potential affinities of the cambroernids in the light of our current knowledge of animal phylogeny is to assess a number of competing hypotheses. In particular, recent animal phylogenies that are based on molecular data continue to provide some reasonably robust topologies of animal groups (e.g., [22], [23]) and in principle can be used to predict the bodyplan of the latest common ancestor of clades. Whilst these phylogenies and predictions are by no means completely consistent they can be used a priori as benchmarks for discussion of the potential affinities of the cambroernids.

### (c) Comparisons to Lophotrochozoans

With respect to the lophotrochozoans an affinity to either the annelids or sipunculans seems unlikely, not least because the tentacles in annelids (notably the serpulids and sabellids) originate from multiple segments. In addition, the sipunculans may well nest within the annelids themselves [24]. However, an important point of reference could be the “Lophophorata” [25] (with an eponymous single bilateral tentacular system comprising a crown of ciliated mesosomal tentacles surrounding the mouth but not the anus). These usually are taken to include the brachiopods, ectoprocts, and phoronids, but in reality their inter-relationships have proved unexpectedly controversial [22], [23], [26]–[30]. Not least this is because the entoprocts, for long effectively an orphan group in phylogenetic limbo, are now proposed by some to be not only lophotrochozoans [22], but close to either the phoronids [30] or the ectoprocts [23]. Other analyses also place the entoprocts, with the ectoprocts, and although tentative identify them as either basal lophotrochozoans [31] or locate them even more remotely within the protostomes [27]. Even within the “classical” lophophorates the proposed positions of the various phyla have varied. For example, the traditional association between the phoronids and brachiopods [26]–[28], [32] is not found in other analyses [23], [29], [30]. A position of the phoronids outside Polyzoa (ectoprocts, entoprocts and cycliophorans)[23] and Kryptrochozoa (including brachiopods), adds to the growing body of work that increasingly suggests that the concept of “lophophorates” is redundant. So too the striking similarity between the tentacular system of the “lophophorates” and the pterobranchs has long been recognized, but in the light of molecular phylogenies these structures are evidently convergent [21], [22].

Any attempt at a comparison between extant lophotrochozoans, notably the tentaculate ectoprocts (and by implication possibly the entoprocts [31]) and the phoronids, and *Herpetogaster* + *Phlogites* will depend not only on the plausibility of identifying homologous structures, but also attempting to choose between the wider and inconsistent phylogenies presented by different researchers. With respect to the phoronids, all of which are all tubicolous (or infaunal), any similarity seems at best superficial. This has particular force with respect to the tentacle structures, as well as a more strongly recurved gut that shows no sense of coiling. There is no obvious equivalent to the stolon or stalk, and in addition there seems to be no reason to equate the anterior four (and possibly five) lobes of *Phlogites* with the single oral epistome of the phoronids. One can also observe that in those phylogenies [26]–[28], [32], admittedly ones that are controversial, that continue to link the phoronids with such groups as the brachiopods (and by implication the annelids and possibly molluscs), the relevance of the cambroernids is not obvious.

In principle, however, a comparison with the entoprocts [33] is more intriguing. Unfortunately their fossil record is very slender and also much younger [34] (although originally compared to the entoprocts the Cambrian *Dinomischus* remains of problematic status [35]), but such similarities as there are with the cambroernids pertain more directly to *Phlogites*. Thus entoprocts also have a stolon and a calyx reminiscent to *Phlogites*, but like phoronids, brachiopods and ectoprocts differ from these fossils by having a single bilateral tentacular system with unbranched tentacles. Their much smaller size could be attributed to miniaturization, but overall the comparisons seem to be too general to carry particular conviction. Brief mention should also be made of the younger *Escumasia*, from the Pennsylvanian Mazon Creek Lagerstätte [36]. Whilst this is also of problematic status, there are some quite striking similarities between these fossils in terms of the arrangement of the calyx and stalk. In the younger form, however, the tentacles are reduced in number and much simpler. Finally given the relationship proposed here between *Herpetogaster* + *Phlogites* and the eldoniids, attention should be drawn to the speculation by Dzik [9] that the latter group are some sort of lophophorates. This notion is based on the possession of tentacles, a U-shaped gut as well as a claim for a marginally secreted external skeleton. Although circular lines in some eldoniids have been interpreted as growth lines [5], [9], they are absent in *Eldonia* itself and whilst marginal lines of successive accretion would not be unexpected, in at least some cases they are more likely to represent compression artefacts of these bell-shaped organisms [5]. Other similarities between the eldoniids and any “lophophorate” seem to be too generalized to carry much phylogenetic weight. In conclusion, whilst a comparison to the entoprocts remains potentially worth entertaining (and even more so if their position within the lophotrochozoans [23], [31] receives further support) we find no compelling reason on present evidence to assign any of these Cambrian fossils to any group within the lophotrochozoans.

### (d) Comparisons to Deuterostomes

#### 1. Comparisons to hemichordates

A possible relationship between *Phlogites* and pterobranchs has been very briefly mentioned elsewhere [37], but contrary to this speculation it is clear that the conspicuous anterior lobes of *Phlogites* cannot be readily equated with the single pre-oral lobe of the pterobranchs. Comparison between any cambroernid and the hemichordates are further complicated because of the obvious disparity between the latter's division into the colonial pterobranchs and vermiform enteropneusts. Thus, cambroernids and hemichordates potentially share a ventral stalk (in enteropneusts) and paired feeding tentacles (in pterobranchs). However, employing these two characters alone would make a hemichordate affinity difficult to support. First, this is because the stalk refers to the ventral post-anal extension of the metacoels in enteropneusts (the “adhesive post-anal tail”). It is only present during early development in *Saccoglossus* and *Spengelia*, and is not found in any other adult forms [38]. A similar structure (the stolon in *Rhabdopleura* and stalk in *Cephalodiscus*) is probably homologous in pterobranchs [38]. As already noted, and in the context of transformations of potential significance, is the fact that the eldoniids do not possess a stalk and this is assumed to have been lost. With respect to the tentacles, if they are indeed homologous, this would imply cambroernids had paired body cavities similar to the left and right mesocoels of extant hemichordates. It is also worth noting that similarly symmetrical coeloms are inferred to have been present in the pre-radial echinoderms (the left and right hydrocoels), suggesting more primitive ambulacrarians in the form of the last common ancestor of echinoderms plus hemichordates would have also possessed such structures (see discussion below).

**Figure 6:**
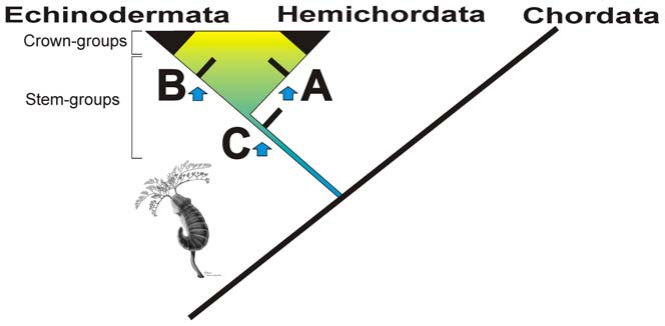
Possible positions of the cambroernids within ambulacrarians. A = stem-group hemichordate, B = stem-group echinoderm, C = stem-group ambulacrarian.

In addition, extant pterobranchs are miniaturized and colonial, live in tubes and their U-shaped gut is probably a consequence of this miniaturization and sessile lifestyle. These characters probably evolved early on in the history of this clade as is evident from the graptolites [39], [40], which date from the earliest Middle Cambrian (including examples from the Burgess Shale [41]). By contrast the cambroernids are much larger, are not colonial and do not form tubes and the presence of a somewhat imperfect U-shaped gut in most forms (but not *Herpetogaster*) might simply be due to convergence. With the exception of an undescribed vermiform species of enteropneusts from the Burgess Shale [42], which suggest that the origin of both hemichordate clades predates the Middle Cambrian, the fossil record is silent regarding the morphology of the early members of this clade. The traditional view that within the hemichordates themselves the pterobranchs are primitive is well entrenched in the literature [38], [43]. An alternative interpretation, of the pterobranchs being derived from within the enteropneust clade, has recently been proposed [44], [45], but this view remains controversial. Using the data currently available, and assuming some sort of relationship the cambroernids do not provide a clear alternative between these two hypotheses. Importantly none of the characteristic features of the hemichordate bodyplan can be unambiguously recognized in these fossils, and at this time a position as a stem-group hemichordate (Fig. 6A) cannot be supported, although in terms of extant forms anatomically *Phlogites* appears to be somewhat closer to a pterobranch-like form.

#### 2. Comparisons to echinoderms

A relationship to crown-group echinoderms has received attention on account of comparisons between *Eldonia* and free swimming holothurians, such as the extant *Pelagothuria natatrix* [8]. Nevertheless although well-known this comparison, let alone to any other extant group of echinoderms [10], [46], [47], remains highly problematic. Thus, *Pelagothuria* has a slender conical body, bears various numbers of webbed podia (from 12 to 16) and the veil is absent on the ventral radius [48]. In detail its morphology, therefore, is quite unlike that of any eldoniid. The presence of rows of pustules on the ventral disc of some eldoniids has been suggested to be homologous to the reduced podia of holothurians [5], [13]. No such structures, however, are present in *Eldonia* and where present the pustules appear to be rather small and dispersed, questioning a functional role as podia. Conceivably, given the relative three-dimensionality of these structures the rows of pustules could represent mineralized sclerites. However, on more general grounds an evolutionary proximity to the holothurians is questionable. This is because whilst their history is mostly based on the distinctive isolated sclerites (and calcareous ring elements) [49] and rare body fossils, both palaeontological data [50] and current phylogenies (both morphological [51] and molecular [52]) place holothurians as a sister group to the echinoids. In addition, both groups evidently represent the most derived of the Echinodermata. Given also that the first holothurians (apodid-like forms) appear in the early Middle Ordovician [53], the stratigraphic record of echinoderms is incongruent with the eldoniids being early members of this group.

We should also note that Chen [13] uses the supposed podia in support of an affinity between the eldoniids and echinoderms, arguing for a position within the basal deuterostomes. Whilst the latter suggestion is broadly consistent with our interpretation, a specific comparison to crown-group echinoderms remains more problematic. This is not only because of the questionable homologies with the eldoniids (and by implication the other cambroernids), but also the relatively good record of Cambrian echinoderms, especially the pre-radial types. These are particularly important because they are thought to retain features also found within the hemichordates, including a post-anal muscular stalk and facultative attachment [43]. The second possibility, therefore, is to enquire whether the cambroernids might still fall within the stem-group echinoderms (Figure 6B). Thus, by subsequently acquiring a mesodermal skeleton (stereom), animals similar to *Herpetogaster* and *Phlogites* would broadly resemble the pre-radial echinoderms in possessing a muscular tail, expanded theca, and conceivably a tentacular extension of a functional hydrovascular system. This scenario, with the cambroernids predating the appearance of the otherwise diagnostic stereom, would also be consistent with the water vascular system (derived from the left hydrocoel only) being not only unique to the echinoderms, but probably appearing amongst the more derived pre-radial echinoderms, most likely the solutes [54].

Unfortunately these ideas are difficult to test in much detail because of: a) important but disputed inferences concerning the soft-bodied anatomy of the pre-radial echinoderms, b) the considerable disparity of this assemblage, and correspondingly c) the controversial nature of their earliest evolution [54]. Thus stylophorans evidently have gill slits, but these are less likely to be phylogenetically informative because such structures may show considerable diversity but still appear to be a deuterostome plesiomorphy [54]. Conceivably the hemispherical to triangular structures in *Herpetogaster* (Figure 3A–E, H) represent openings from the pharynx, and as such could qualify as pharyngeal openings serving as gills. No equivalent, however, can be identified in the other cambroernids, suggesting that if indeed some taxa possessed gill slits they were lost in most, if not all, members of this group. Given gill slits are evidently plesiomorphic to the deuterostomes they must have been present amongst the primitive ambulacrarians. They occur in hemichordates, and are also reliably identified in the stylophorans. In the cinctans, if present at all, they may have opened into an anterior pharynx [54], [55]. They are less obvious in the other pre-radial echinoderms, and within the echinoderms they were evidently lost at least once.

Importantly, however, in the context of a possible relationship to the cambroernids, the cinctans are hypothesized to have possessed a water vascular system with right and left-hand hydrocoels presumably extending into two tentacular elements of unequal size [54], [56]. The function of these inferred tentacles was probably connected to feeding; their position could have been exothecal or when inferred from skeletal impressions confined within the paired anterior marginal grooves [55]. Since the right marginal groove is always the shortest (or interestingly is absent in some forms), this suggests at least an incipient reduction of the right-hand hydrocoel by torsion [55] with presumably the loss of an associated tentacle if originally present. In this evolutionary scenario the next stage would be the final loss of the tentacles. This presumably occurred in the solutans, where they are replaced with a feeding arm (ambulacrum), so indicating the evolution of the diagnostic water vascular system. Ctenocystoids are somewhat similar to cinctans but with a straight gut and a pair of bilaterally anterior feeding grooves. Ctenocystoids are thought to be more primitive than cinctans but still located within the stem lineage echinoderms and to approximate to the hypothetical ambulacrarian ancestor [54]. In this schema the cambroernids (possessing bilaterally symmetrical tentacles that suggest the presence of a paired coelomic cavity) could presumably be placed stemward of the cinctans and ctenocystoids, evolving before the evolution of the stereom and yet closer to the hypothetical ambulacrarian ancestor.

#### 3. Comparisons to stem-group ambulacrarians

Based on molecular phylogenies, fossils and detailed developmental studies, the last common ancestor of hemichordates and echinoderms is predicted to have been bilaterally symmetrical, a filter feeder with a paired water vascular system constructed from right and left hydrocoels, and with a muscular post-anal stalk ([43] see references herein). In addition this animal would have been equipped with a filter feeding pharynx [39]). In this context, the cambroernids might represent the earliest ambulacrarians and thus be our best clue as to the emergence of the ambulacrarian bodyplan (Fig. 6C).

Any discussion of the stem-group ambulacrarians must also in principle consider the somewhat problematic xenoturbellans, a group of modern free-living worm-like animals. In *Xenoturbella* the animal shows a very simple organisation, with a diffuse nervous system and apart from a statocyst lacks defined organs. Since its discovery 50 years ago numerous proposals concerning its phylogenetic position have been put forward [57], although recent molecular data argue (as well as earlier morphological studies (e.g., [58]) for a position as a stem-group ambulacrarian [22], [45], [59] probably closer to hemichordates [60]. However, other molecular studies do not entirely agree with this interpretation [61]. These studies, together with the fact that the xenoturbellids appear to have very low number of hox-genes [62], suggest that they may be more basal deuterostomes than previously thought, while the likelihood of a yet more primitive position [63] is now receiving fresh new support [23]. In any event it is evident that irrespective of their still controversial phylogenetic position the cambroernids cannot throw useful light on the xenoturbellid question.

### (e) Evolutionary Implications

Arriving at a precise phylogenetic position for the cambroernids, therefore, has proved difficult. On balance a place amongst the tentaculate lophotrochozoans seems to be less persuasive. Given a place within the ecdysozoans is even less plausible, then the final possibility must be to look to the deuterostomes. Here, as noted the options revolve around a series of possibilities, including a stem-group echinoderm, a hemichordate or an ambulacrarian. Whilst this list of possibilities might seem to leave the matter largely unconstrained, it is important to stress that from a Cambrian perspective the morphological differences between these various alternatives were probably insignificant. If, for the sake of the argument, the position of the cambroernids does indeed lie near the branching point of the two main ambulacrarian clades that led ultimately to the echinoderms and hemichordates, then we should not be surprised that it seems reminiscent of both pterobranchs and pre-radial echinoderms.

Finally, if accepted as some sort of deuterostome then these fossils have some further interesting implications. For example, consider the possible segmentation in *Herpetogaster* (Figure 3A, C, D, F, H). In overall form this is intriguingly reminiscent of the dorsal segmentation seen in the trunk of the Cambrian yunnanozoans [64]–[67]. Although yunnanozoans are vaguely fish-like, and have been allied to the chordates [66], [67], an alternative view has placed them amongst the hemichordates [64], [65], [68], if not yet more primitive deuterostomes [65], [68]. If the primitive deuterostomes were indeed segmented and free living, then the cambroernids could be particularly instructive with respect to the early evolution of the Ambulacraria. Thus, one of the key steps in their evolution was arguably the adoption of initially a semi-sessile mode of life. This was marked by acquisition of prominent tentacles for suspension feeding, a reduction of the head region with a corresponding re-organization of the coelomic cavities, and ultimately the loss of segmentation (albeit partially retained in *Herpetogaster*). It is at this juncture that we predict (see also [43]) the dichotomy towards the first echinoderms (acquiring stereom and ultimately a water vascular system) and the hemichordates. Whilst many of the evolutionary steps involved in this process are still hypothetical, we suggest that animals similar to *Herpetogaster* may, in terms of the fossil record, be our best current glimpse of a very primitive ambulacrarian. In addition, if the cambroernids are confirmed as a group of Palaeozoic ambulacrarians, a long-standing evolutionary question is resolved that in addition suggests a hitherto unappreciated diversity of form and ecology amongst the ambulacrarians, not least in the form of the medusiform eldoniids.

## Materials and Methods

Most specimens of *Herpetogaster* were collected *in-situ* by Royal Ontario Museum field crews. When possible, specimens were prepared to remove rock-coated parts using an engraving tool, along with a thin reamer made of tungsten carbide. Camera lucida drawings were produced (with both part and counterpart) when available to represent some particular morphological features that are difficult to see on photographs. Pictures were taken by applying polarizing filters to both the camera and the light-source in order to increase contrast on wet or dry specimens. Scanning electron microscope (SEM) photographs of uncoated specimens were taken at McMaster University using a FEI-Electroscan ESEM 2020 microscope. Basic measurements (Figure S1) were taken from live digital images using ECLIPSE NET 1.2 and Nis-Elements D 2.3 from a Nikon DS-5M digital camera mounted on a Nikon SMZ-1500 stereoscopic microscope at the Royal Ontario Museum.

### Nomenclatural Acts

The electronic version of this document does not represent a published work according to the International Code of Zoological Nomenclature (ICZN), and hence the nomenclatural acts contained in the electronic version are not available under that Code from the electronic edition. Therefore, a separate edition of this document was produced by a method that assures numerous identical and durable copies, and those copies were simultaneously obtainable (from the publication date noted on the first page of this article) for the purpose of providing a public and permanent scientific record, in accordance with Article 8.1 of the Code. The separate print-only edition is available on request from PLoS by sending a request to PLoS ONE, 185 Berry Street, Suite 3100, San Francisco, CA 94107, USA along with a check for $10 (to cover printing and postage) payable to “Public Library of Science”.

In addition, this published work and the nomenclatural acts it contains have been registered in ZooBank, the proposed online registration system for the ICZN. The ZooBank LSIDs (Life Science Identifiers) can be resolved and the associated information viewed through any standard web browser by appending the LSID to the prefix “http://zoobank.org/”. The LSID for this publication is:urn:lsid:zoobank.org:pub:502400AC-FD1B-41FA-AAE4-683089B7C2EA

Digital Archiving: PubMedCentral and LOCKSS

## Supporting Information

Figure S1 A, camera lucida of the holotype of *Herpetogaster collinsi* showing the different body parts that have been measured on dorso-ventrally preserved specimens. B, summary results of measurements, basic statistics and observations based on A. C, length-to-width ratio for different body zones suggesting isometric growth.
(4.65 MB TIF)

Figure S2 *Herpetogaster collinsi* from the Middle Cambrian Burgess Shale. A–D, ROM 58084, cluster of specimens mixed with sponges including *Vauxia* sp. and *Hazelia* sp.; A, overall view, image in cross-nicols; B, overall view, image with high angle of light, arrows indicate specimens of *Herpetogaster*; C, close-up of the framed area in a, detail of one specimen showing the stolon possibly inserted within the oscula of *Vauxia* sp.; D, close-up of the framed area in C. E–F, specimen ROM 58058 with the terminal disk at the end of the stolon attached to *Vauxia* sp., the stolon is rotated 180 degree presumably as a result of burial; E, overall view; F, close-up of the framed area in E. Scale bars: A,B, 20 mm; C,E, 10 mm; D,F, 1 mm. va = *Vauxia*, hz = *Hazelia*. Legend as in Figure 1.
(12.54 MB TIF)

Figure S3 Camera-lucida drawings of *Phlogites longus* from the Lower Cambrian Chengjiang biota. A, ELI-Phl-07-001. B, ELI-Phl-07-003. C, ELI-Phl-07-002. Scale bars: 5 mm. Legend, an, anus; in, intestine; lo, lobe; ro?, reproductive organs?; st, stolon; stom, stomach; te, tentacle; tr, trilobite fragment.
(8.92 MB TIF)
