# Supplementary figures and images for: Tentaculate Fossils from the Cambrian of Canada (British Columbia) and China (Yunnan) Interpreted as Primitive Deuterostomes
Source: PLoS One. 2010 Mar 8;5(3):e9586. doi: 10.1371/journal.pone.0009586 (PMC2833208; doi:10.1371/journal.pone.0009586)

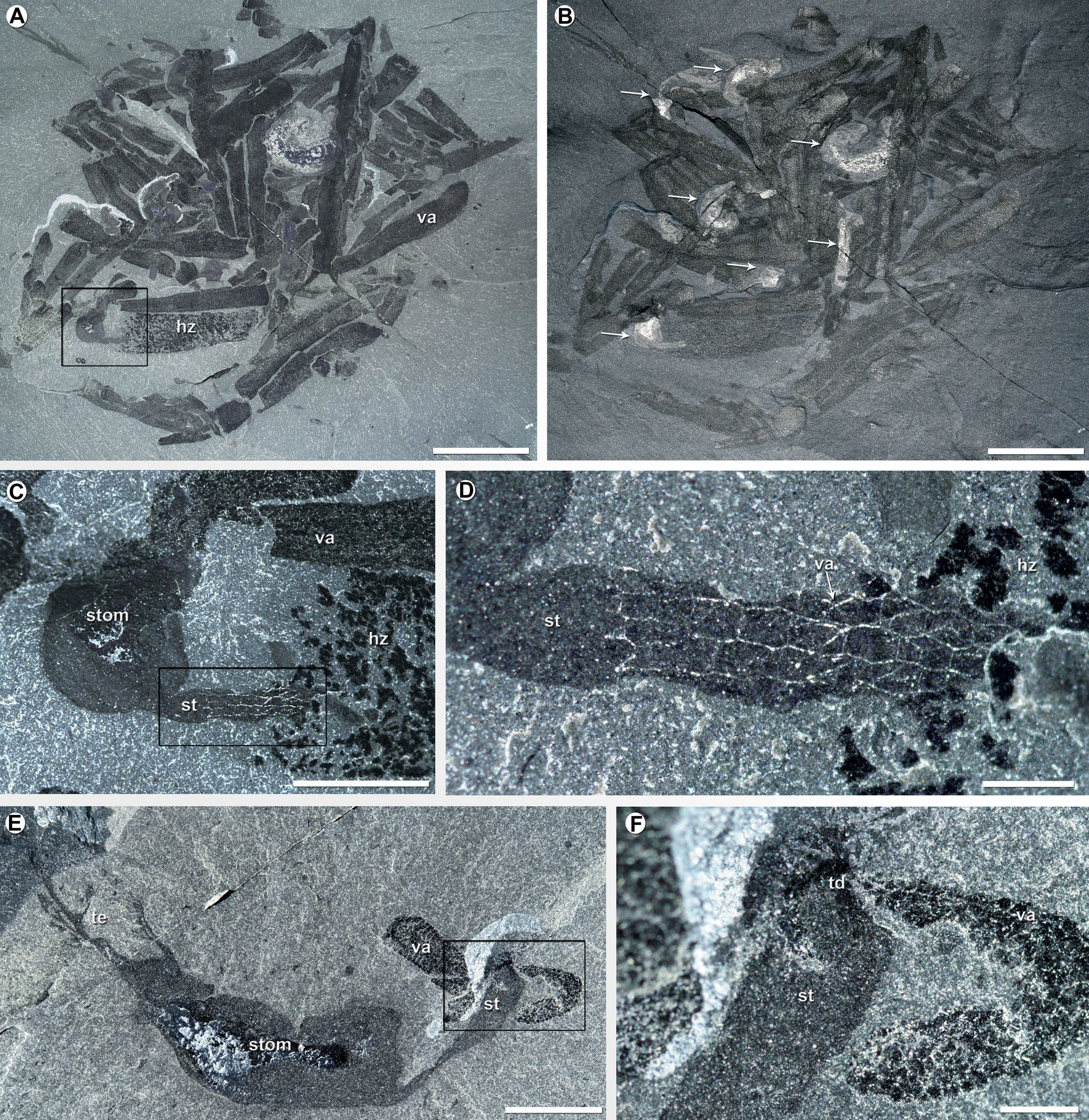

Supplement: Figure S2 — Herpetogaster collinsi from the Middle Cambrian Burgess Shale. A–D, ROM 58084, cluster of specimens mixed with sponges including Vauxia sp. and Hazelia sp.; A, overall view, image in cross-nicols; B, overall view, image with high angle of light, arrows indicate specimens of Herpetogaster; C, close-up of the framed area in a, detail of one specimen showing the stolon possibly inserted within the oscula of Vauxia sp.; D, close-up of the framed area in C. E–F, specimen ROM 58058 with the terminal disk at the end of the stolon attached to Vauxia sp., the stolon is rotated 180 degree presumably as a result of burial; E, overall view; F, close-up of the framed area in E. Scale bars: A,B, 20 mm; C,E, 10 mm; D,F, 1 mm. va = Vauxia, hz = Hazelia. Legend as in Figure 1. (12.54 MB TIF) [file pone.0009586.s002.tif]

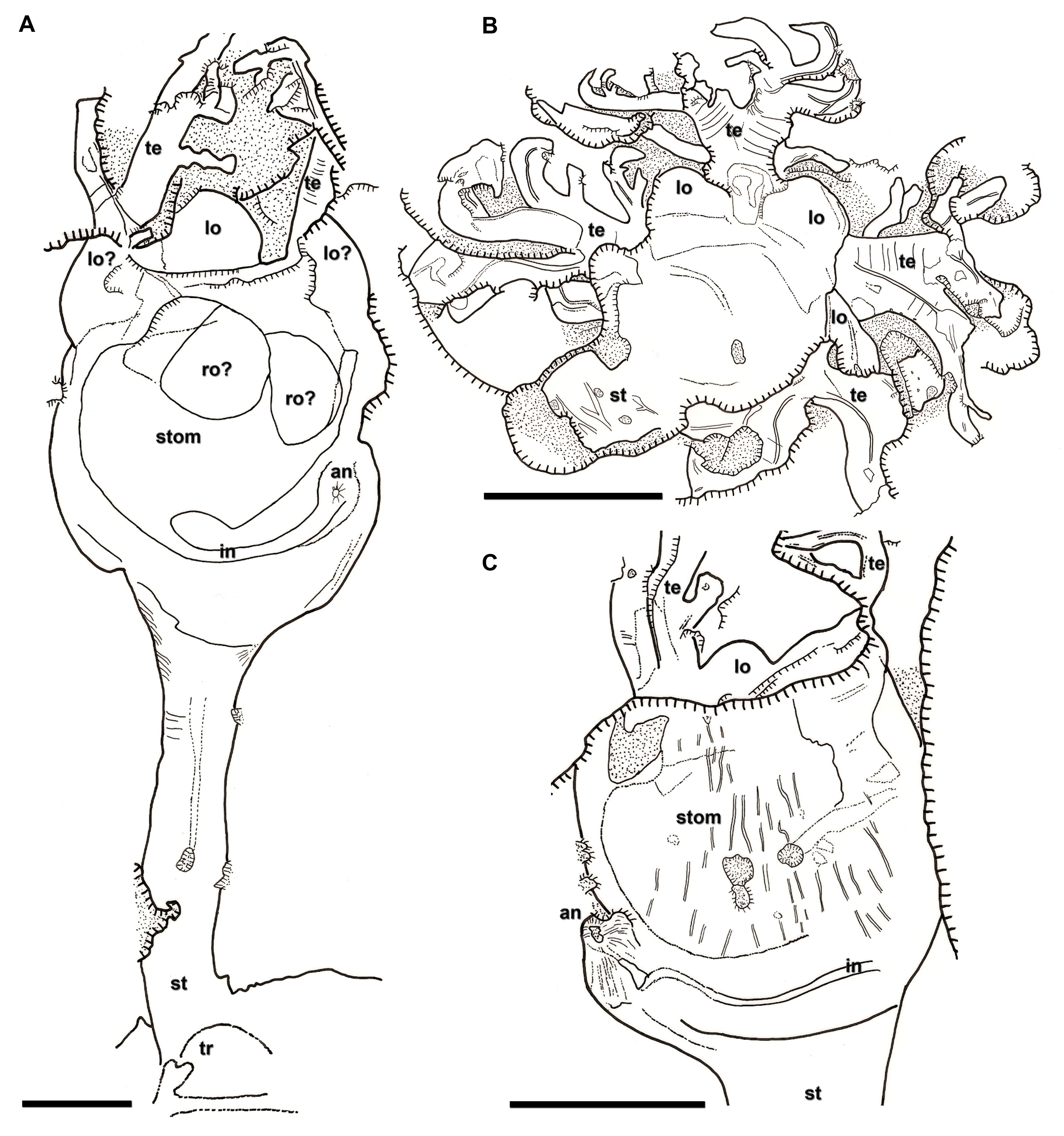

Supplement: Figure S3 — Camera-lucida drawings of Phlogites longus from the Lower Cambrian Chengjiang biota. A, ELI-Phl-07-001. B, ELI-Phl-07-003. C, ELI-Phl-07-002. Scale bars: 5 mm. Legend, an, anus; in, intestine; lo, lobe; ro?, reproductive organs?; st, stolon; stom, stomach; te, tentacle; tr, trilobite fragment. (8.92 MB TIF) [file pone.0009586.s003.tif]
